# Virological Events During Immune Checkpoint Inhibitor Therapy: A Multi-Source Evaluation

**DOI:** 10.3390/ph19101616

**Published:** 2026-10-12

**Authors:** Yue Cao, Zenghui Zhang, Yanying Liang, Chenxi Zhao, Xvrong Li, Qingluan Yang, Lingyun Shao

**Affiliations:** 1 Department of Infectious Diseases, Shanghai Key Laboratory of Infectious Diseases and Biosafety Emergency Response, National Medical Center for Infectious Diseases, Huashan Hospital, Fudan University, Shanghai 200040, China; 17301050072@fudan.edu.cn (Y.C.); zzh19301050120@163.com (Z.Z.); 20301050123@fudan.edu.cn (Y.L.); zhaocx20@fudan.edu.cn (C.Z.); 19301050236@fudan.edu.cn (X.L.); 2 Shanghai Sci-Tech Inno Center for Infection & Immunity, Shanghai 200438, China; 3 Key Laboratory of Medical Molecular Virology (MOE/MOH), Shanghai Medical College, Fudan University, Shanghai 200032, China

**Keywords:** immune checkpoint inhibitors, viral reactivation, chronic viral infections, latent viral infections, immune-related adverse events, pharmacovigilance, FAERS

## Abstract

**Background/Objectives:** Virological findings during immune checkpoint inhibitor (ICI) therapy may represent viral loss of control, pre-existing infection, tumour biomarkers, or infection after immune-related adverse event (irAE)-directed immunosuppression. Distinguishing these possibilities is important for treatment and diagnostic decisions. We evaluated virus-specific patterns across complementary data sources. **Methods:** We evaluated HBV, HCV, HIV, EBV, CMV, HSV, and VZV using FAERS disproportionality analysis (2011Q1–2026Q1), PubMed evidence mapping and appraisal, EBV endpoint verification, and review of ClinicalTrials.gov protocols by start era. Findings were interpreted in relation to prevention, monitoring, endpoint interpretation, and infectious mimic evaluation. **Results:** Among 200,699 primary suspect ICI reports, only HBV reactivation met the prespecified reporting criterion (226 reports; reporting odds ratio 1.20, 95% CI 1.06–1.37); this estimate did not persist with an oncology-restricted comparator (0.81, 0.71–0.94). Same report overlap of HBV with hepatic events and CMV with enterocolitis or pneumonitis identified diagnostically ambiguous settings without establishing event sequence. EBV DNA reflected viral inflammatory activity in some conditions but tumour burden in EBV-associated cancers. The evidence map included 198 publications, with design-specific appraisal of 84. Among 164 trials, conditional viral eligibility appeared in 49.0–57.1% across start eras, whereas longitudinal monitoring appeared in 8.2–19.6%. **Conclusions:** Reporting patterns were virus-specific and comparator-sensitive. The findings support HBV prevention, confirmation of HCV and HIV control, disease-specific interpretation of EBV markers, and CMV evaluation when inflammation or irAE-directed immunosuppression raises concern for infection. Context-specific assessment may help avoid unnecessary ICI interruption while identifying viral loss of control and infectious mimics.

## 1. Introduction

Immune checkpoint inhibitors (ICIs) have become integral to the treatment of a broad range of malignancies by releasing inhibitory immune pathways and restoring antitumour T cell activity [1,2]. Their expanding use increasingly includes patients with chronic or latent viral infections, in whom virological findings may influence decisions regarding ICI continuation, antiviral treatment, immunosuppression, and diagnostic investigation. The background burden is clinically substantial but varies by geography and patient population. In a prospective multicentre cohort of 3051 patients with newly diagnosed cancer, previous HBV infection, chronic HBV infection, HCV infection, and HIV infection were detected in 6.5%, 0.6%, 2.4%, and 1.1%, respectively; exposure to latent herpesviruses is substantially more widespread, with an estimated global CMV seroprevalence of 83% in the general population [3,4]. These estimates describe different epidemiological states and should not be interpreted as the incidence of viral reactivation or disease during cancer therapy. Checkpoint blockade can reinvigorate selected antiviral T cell responses during chronic infection [5,6]. This effect, however, is neither uniform nor equivalent to complete restoration of host defence. Responses to PD-1 blockade arise predominantly from progenitor-like exhausted T cell subsets, depend on competent antigen presentation and co-stimulatory signalling, and may remain constrained by persistent antigen exposure and stable epigenetic programmes of exhaustion [7,8,9]. Dendritic cell–T cell interactions further shape the balance among pathogen control, immune tolerance, and inflammatory tissue injury [10]. Virological findings during ICI therapy therefore arise within an immunological setting that can support antiviral activity, inflammatory injury, or both.

The clinical interpretation of these events is further complicated by the dynamic interaction between immune-mediated toxicity, tissue injury, and its treatment. Immune-related adverse events (irAEs) can disrupt epithelial barriers and produce organ manifestations that overlap with viral disease, particularly in the liver, gastrointestinal tract, and lungs. Treatment of moderate or severe irAEs may require high-dose corticosteroids, tumour necrosis factor inhibitors, or other immunosuppressive agents, creating a time-dependent pathway to opportunistic infection even when baseline viral infection was controlled [11,12,13,14]. Infection and immune-mediated inflammation may also coexist: viral detection does not establish that the virus caused the organ injury, whereas a presumptive irAE diagnosis does not exclude concurrent infection. Concomitant anticancer therapy, advanced malignancy, baseline organ dysfunction, and differences in virological surveillance further influence the occurrence, detection, and reporting of these events. This distinction is clinically consequential because misclassifying infection as an irAE may intensify immunosuppression, whereas misclassifying an irAE as infection may delay appropriate treatment or prompt unnecessary ICI interruption. Virological findings must therefore be interpreted in relation to tissue involvement, treatment exposure, and event chronology rather than in isolation.

We evaluated seven viral contexts because they differ in their biology of persistence, routine surveillance, and the clinical meaning of virological detection. HBV persists through intrahepatic covalently closed circular DNA and has an established framework for screening, prophylaxis, and reactivation [15,16,17]. HCV is not managed through an analogous latent reactivation paradigm after sustained virological response; detectable HCV RNA instead indicates current viraemia, relapse, or reinfection [18,19]. HIV establishes a persistent reservoir, but its interpretation during cancer therapy centres on antiretroviral continuity, virological suppression, and immune status [20,21,22]. The herpesviruses establish lifelong latency but raise virus- and tissue-specific questions: EBV DNA may reflect viral inflammatory disease activity or tumour burden [23,24], CMV detection may require differentiation between asymptomatic replication and tissue-invasive disease that mimics an irAE [11], and HSV or VZV events may be particularly sensitive to clinical recognition and coding definitions. A positive viral assay, a reported infection, and clinically meaningful loss of viral control are therefore not interchangeable outcomes.

Evidence is scattered across spontaneous reports, clinical studies, and trial protocols, where terms such as infection and reactivation may describe biologically different outcomes. No single source can fully distinguish a reporting signal from altered viral control, a disease- or tumour-related biomarker, or an infection that overlaps with immune-mediated toxicity. We therefore conducted a multi-source evaluation in which each source addressed a different part of this problem. FAERS characterized virus-specific reporting disproportionality, organ domain overlap, and robustness across analytic definitions. PubMed evidence mapping and design-specific appraisal characterized the clinical literature; verified EBV studies clarified endpoint meaning; and ClinicalTrials.gov analyses examined viral eligibility, prophylaxis, and monitoring criteria across trial start eras. By keeping these evidence layers analytically distinct, we sought to clarify which findings warrant prevention or further investigation, which primarily serve as monitoring or tumour biomarkers, and when a controlled chronic or latent viral background may not by itself warrant ICI interruption.

## 2. Results

### 2.1. Analytic Datasets and Evidence Coverage

**Figure 1:**
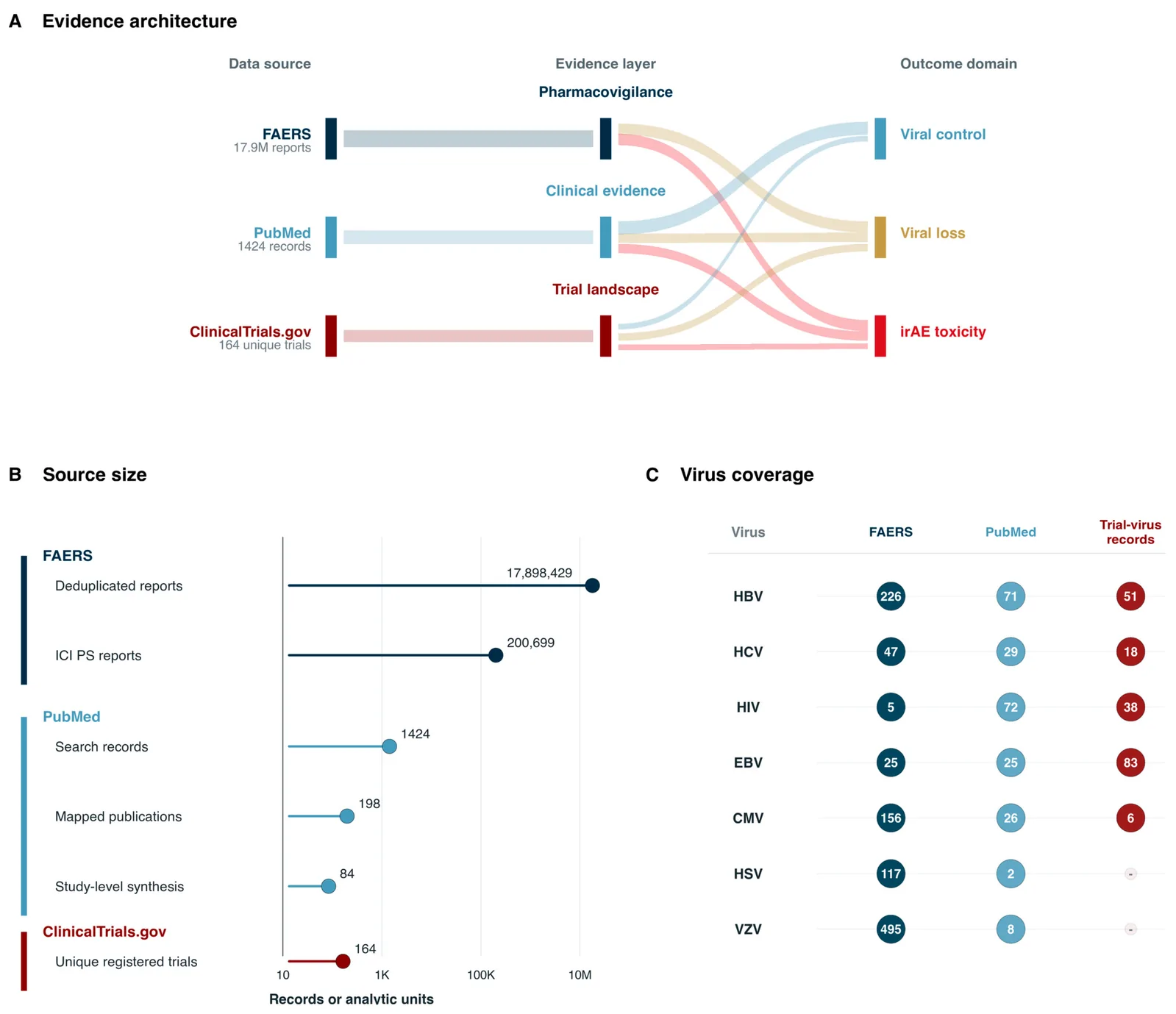
Multi-source analytic framework and evidence coverage. (**A**) Analytic framework linking the FDA Adverse Event Reporting System (FAERS), PubMed evidence mapping, and the ClinicalTrials.gov trial landscape to viral control, viral loss, and immune-related adverse event (irAE) domains. Ribbon widths are schematic and do not represent evidence weight. (**B**) Source-specific counts of deduplicated FAERS reports, primary suspect immune checkpoint inhibitor (ICI) reports, PubMed search records, mapped publications, publications in the study-level evidence set, and unique registered trials. (**C**) Coverage of the seven viral contexts across the three sources. Values represent ICI reports containing the specified viral outcome, mapped publications, and trial–virus records. Panels A and B use unique trial counts, whereas panel C uses trial–virus records; one trial may contribute to more than one viral context. Multivirus publications may likewise contribute to more than one row. Circle size is constant, and a dash indicates that the viral context was outside the registry analysis scope. Source-specific counts have different units and are not directly comparable. FAERS counts represent reports rather than patients, and registry counts describe protocol handling rather than clinical outcomes.

The analytic framework comprised pharmacovigilance data, mapped clinical literature, focused EBV endpoint verification within the literature set, and trial protocol evidence (Figure 1A). FAERS included 17,898,429 deduplicated reports, of which 200,699 listed an ICI as the primary suspect. The PubMed evidence map contained 198 unique publications: 84 entered the study-level evidence set, whereas 114 contributed only to descriptive evidence volume mapping (Supplementary Table S1). ClinicalTrials.gov contributed 196 trial–virus records from 164 unique trials (Figure 1B).

Coverage varied according to the analytic scope of each source, with broader representation of the seven viral contexts in FAERS and PubMed than in the protocol-level registry mapping (Figure 1C). Design-specific appraisal was completed for all 84 study-level records. Framework-specific judgements comprised one “include,” 77 “include with caution,” 4 “some concerns,” 1 “include with major limitations,” and 1 “serious risk of bias” (Supplementary Table S2). Because these judgements originated from different design-specific tools, they were not interpreted as a common ordinal scale. A malignant cancer indication was mapped in 171,592 of the 200,699 ICI reports; incomplete and heterogeneous indication coding precluded cancer-specific risk estimation (Supplementary Figure S1). The populations, analytic roles, and inferential scope of all data sources are summarized in Table 1.

### 2.2. HBV Was the Only Viral Outcome That Met the Overall FAERS Reporting Criterion

**Figure 2:**
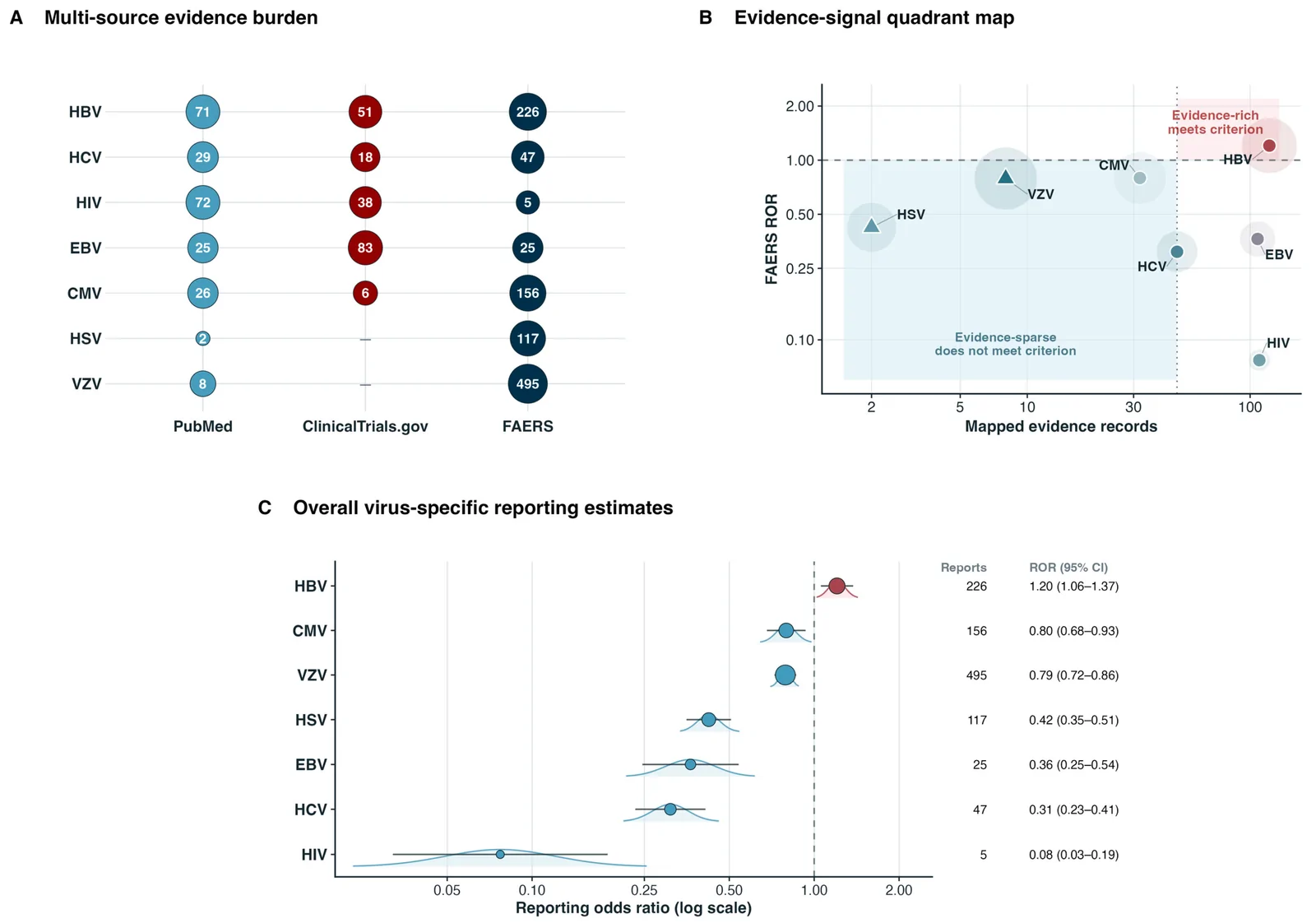
Virus-specific evidence distribution and FAERS reporting estimates. (**A**) Source-specific counts for the seven viral contexts. Values represent mapped PubMed publications, ClinicalTrials.gov trial–virus records, and primary suspect ICI reports containing the corresponding viral outcome in FAERS. Circle area is scaled to the log-transformed count. A dash indicates that the viral context was outside the registry analysis scope. Counts have source-specific units and should not be compared as equivalent observations. (**B**) Overall FAERS reporting odds ratio (ROR) plotted against the combined number of mapped PubMed publications and ClinicalTrials.gov trial–virus records. Halo area represents the number of ICI reports containing the viral outcome. Circles indicate mapping in both contextual sources; triangles denote PubMed-only mapping for viruses outside the registry scope. The dashed horizontal line marks ROR = 1, and the dotted vertical line marks the median mapped evidence count. Shaded regions are descriptive and do not represent evidence grades, clinical priorities, or causal classifications. (**C**) Overall RORs and 95% confidence intervals for the seven viral outcomes among primary suspect ICI reports compared with all other deduplicated FAERS reports. Point area represents report count, and ridge shapes visually approximate uncertainty around log(ROR). The prespecified reporting criterion required at least three exposed event reports and a lower 95% confidence limit above 1.0. Evidence counts describe volume rather than strength; FAERS estimates describe reporting disproportionality rather than incidence, treatment effects, or causality, and RORs below 1.0 do not imply protection.

The distribution of records differed across data sources. PubMed publications were most numerous for HIV and HBV, ClinicalTrials.gov records for EBV and HBV, and FAERS reports for VZV, HBV, and CMV (Figure 2A). Evidence volume and FAERS reporting estimates showed different distributions: HIV and EBV had relatively large numbers of mapped records but RORs below 1, whereas the HBV estimate was above 1 (Figure 2B).

Among 200,699 primary suspect ICI reports, HBV reactivation was the only one of the seven broad viral outcomes to meet the prespecified reporting criterion (226 reports; ROR 1.20, 95% CI 1.06–1.37). None of the other six viral outcomes met the criterion (Figure 2C; Supplementary Table S3). RORs quantify relative reporting disproportionality within FAERS and do not estimate incidence, comparative risk, or a causal effect of ICI exposure. Estimates below 1 were therefore not interpreted as protective effects.

### 2.3. Immune–Inflammatory Reporting Differed by Organ Domain

**Figure 3:**
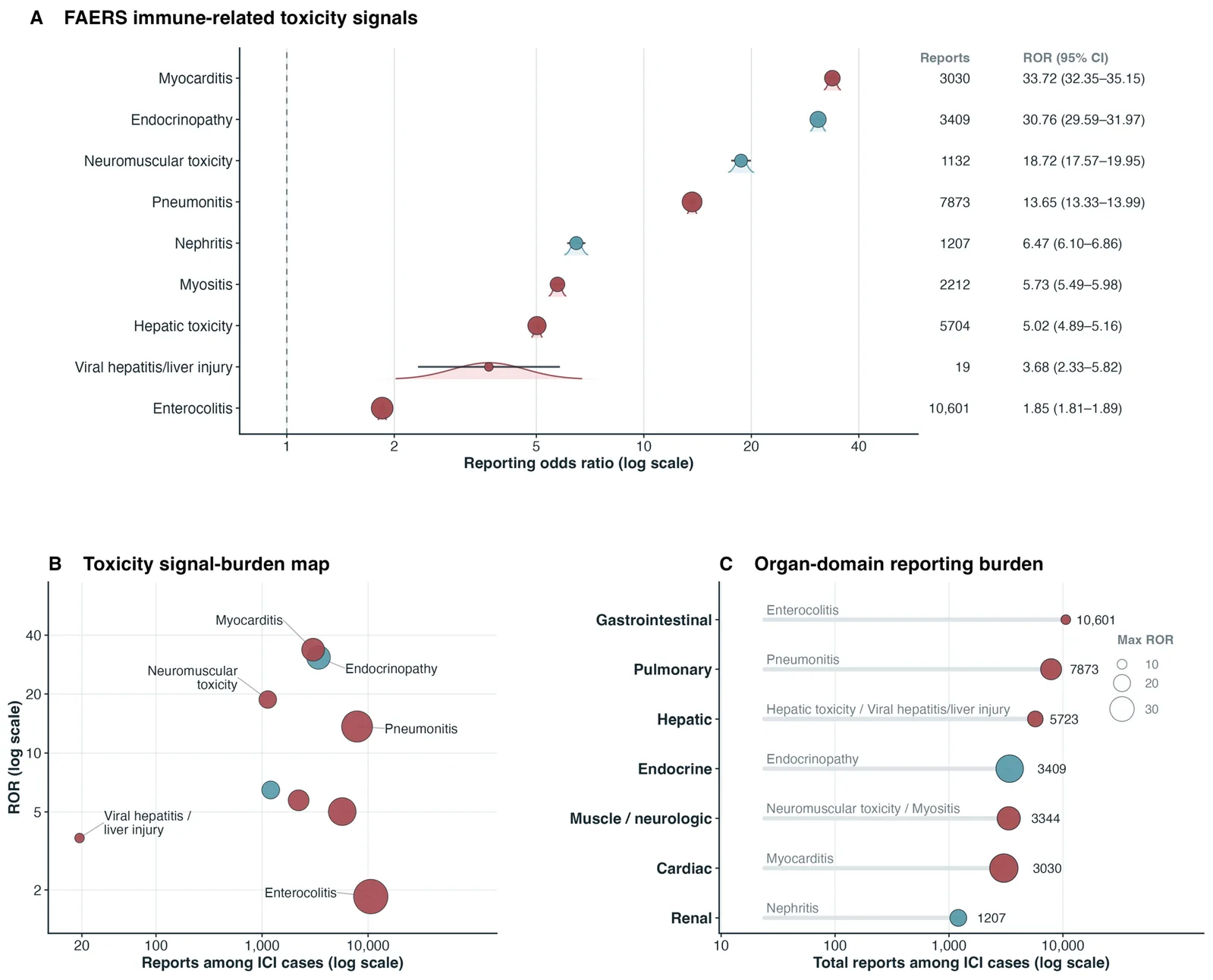
Immune–inflammatory toxicity reporting landscape during ICI therapy. (**A**) Reporting odds ratios (RORs) and 95% confidence intervals for prespecified immune–inflammatory outcomes in 181,500 reports listing a PD-1 or PD-L1 inhibitor as the primary suspect, compared with all other deduplicated FAERS reports. Point area represents report count, and ridge shapes visually approximate uncertainty around log(ROR). (**B**) Relationship between report count and ROR for the same outcomes; point area represents report count. (**C**) Aggregation by organ domain. Horizontal position and adjacent values represent total domain-specific report counts, whereas point area represents the highest ROR among outcomes assigned to that domain. Red identifies prespecified domains in which immune-mediated inflammation may overlap clinically with infectious or viral syndromes; blue identifies other immune-related domains. The colours indicate diagnostic context rather than viral activation or mechanism. The viral hepatitis/liver injury category reflects reported adverse event terminology and does not confirm viral hepatitis. FAERS estimates describe reporting disproportionality rather than incidence or causality.

A complementary analysis examined 181,500 reports listing a PD-1 or PD-L1 inhibitor as the primary suspect. All nine prespecified immune–inflammatory outcome groups were disproportionately reported. RORs were highest for myocarditis (33.72, 95% CI 32.35–35.15), endocrinopathy (30.76, 29.59–31.97), neuromuscular toxicity (18.72, 17.57–19.95), and pneumonitis (13.65, 13.33–13.99) (Figure 3A). Report counts showed a different ordering, with enterocolitis (10,601 reports) and pneumonitis (7873 reports) accounting for the largest numbers of reports (Figure 3B). At the organ domain level, gastrointestinal and pulmonary events accounted for the largest numbers of reports, whereas the highest RORs occurred in the cardiac, endocrine, and muscle/neurological domains (Figure 3C; Supplementary Table S4).

### 2.4. Virus–Organ Same Report Overlap Differed Across Viral Contexts

**Figure 4:**
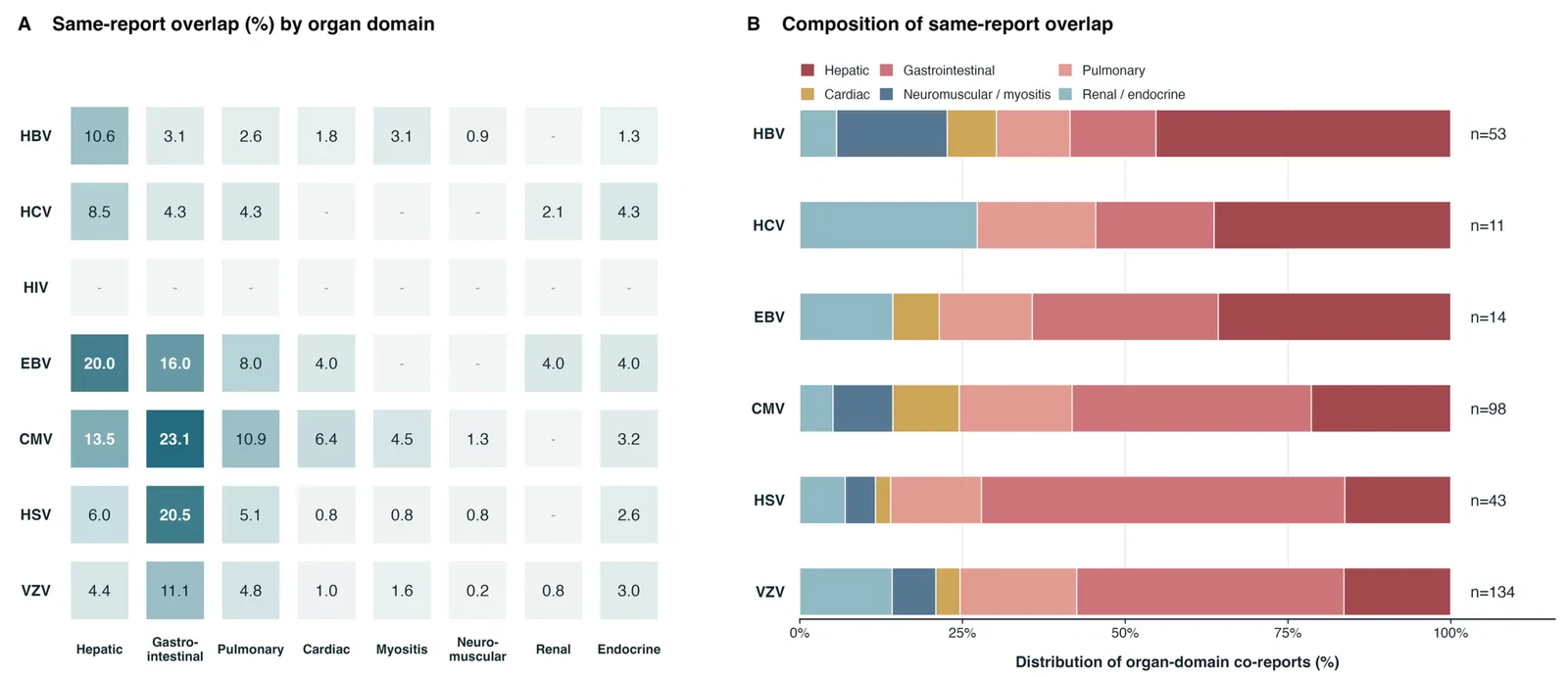
Virus–organ same report overlap. (**A**) Percentage of primary suspect ICI reports with each viral outcome that also contained the indicated organ domain outcome. Cell shading and printed values represent the same percentage; a dash indicates no observed overlap. (**B**) Distribution of observed same report overlaps across six display domains. Myositis and neuromuscular toxicity are combined, as are renal and endocrine outcomes. The adjacent *n* is the sum of domain-specific overlaps, not the number of unique reports or patients. HIV is omitted because none of the five HIV reports contained a prespecified organ domain outcome. Organ domains are not mutually exclusive, and one report may contribute to more than one cell or segment. These descriptive analyses do not establish incidence, event sequence, infection after irAE-directed immunosuppression, mechanism, or causality.

Same report overlap varied across viral contexts and organ domains (Figure 4A; Supplementary Table S5). Gastrointestinal overlap was most frequent for CMV (36/156, 23.1%) and HSV (24/117, 20.5%), whereas hepatic overlap was most frequent for EBV (5/25, 20.0%) and CMV (21/156, 13.5%). In focused analyses, hepatic events were present in 24 of 226 HBV reports (10.6%), whereas 48 of 156 CMV reports (30.8%) contained enterocolitis, pneumonitis, or both.

Among virus–organ combinations with at least one overlapping report, hepatic events represented the largest proportion for HBV, HCV, and EBV, whereas gastrointestinal events predominated for CMV, HSV, and VZV (Figure 4B). None of the five HIV reports contained a prespecified organ domain event. A separate indication mapping audit described cancer group composition and coding completeness but did not provide cancer-specific denominators (Supplementary Table S5). These findings identify viral and inflammatory terms recorded within the same report but do not establish their temporal or causal relationship.

### 2.5. Virus-Specific Estimates Varied Across Sensitivity Analyses

**Figure 5:**
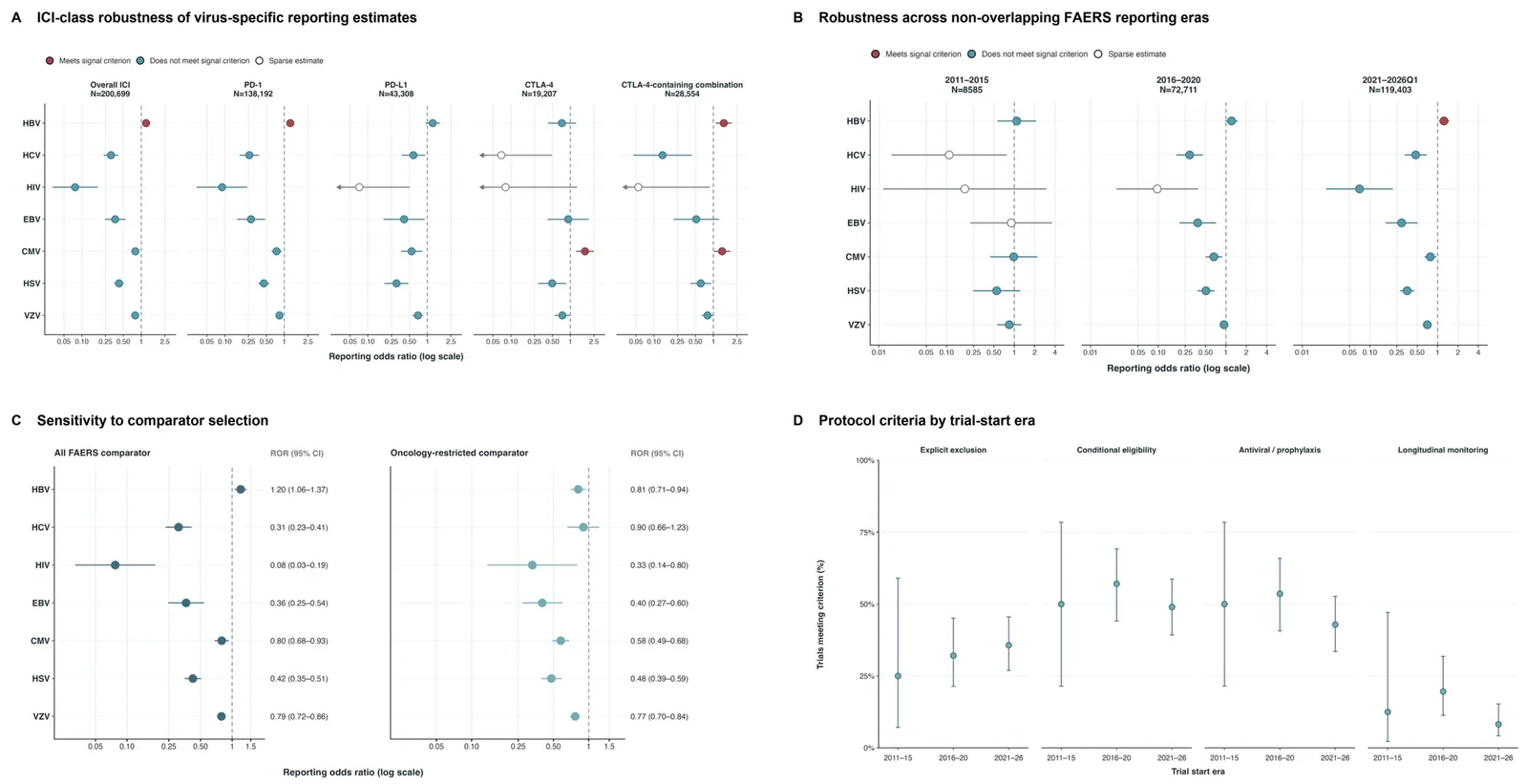
Robustness and sensitivity analyses. (**A**) Virus-specific reporting odds ratios (RORs) and 95% confidence intervals in the overall primary suspect ICI cohort and exploratory PD-1, PD-L1, CTLA-4, and CTLA-4-containing combination groups. The combination definition allowed relevant agents recorded as primary or secondary suspects. Exposure groups overlap and are not direct treatment comparisons. (**B**) Virus-specific estimates in three non-overlapping FAERS reporting eras: 2011–2015, 2016–2020, and 2021–2026Q1. In panels A and B, filled red points meet the prespecified reporting criterion, filled blue points do not, and hollow points denote sparse estimates. Horizontal lines are 95% confidence intervals; arrows indicate intervals extending beyond the displayed axis. (**C**) Sensitivity to comparator selection. The left analysis uses all other deduplicated FAERS reports; the right restricts the comparison to malignant cancer indication reports without recognized ICI primary or secondary suspect exposure. Printed values are RORs with 95% confidence intervals. (**D**) Proportions of unique ClinicalTrials.gov trials meeting four protocol criteria, stratified by trial start era. Points represent observed proportions and vertical bars represent 95% Wilson confidence intervals. Era denominators were 8 trials in 2011–2015, 56 in 2016–2020, and 98 in 2021–2026; two pre-2011 trials were retained in the overall mapping but not displayed. FAERS sensitivity analyses assess the stability of reporting patterns rather than incidence, comparative treatment risk, or causality; RORs below 1.0 do not imply protection. Registry estimates describe protocol language rather than patient outcomes.

Sensitivity analyses examined variation by ICI exposure group, reporting era, comparator population, and outcome definition. In the exposure group analysis, only HBV and CMV met the reporting criterion in at least one subgroup. HBV met the criterion in the PD-1 group (ROR 1.26, 95% CI 1.08–1.47) and the CTLA-4-containing combination group (1.50, 1.10–2.04); CMV met the criterion in the CTLA-4 group (1.77, 1.26–2.49) and the CTLA-4-containing combination group (1.40, 1.03–1.92) (Figure 5A; Supplementary Table S3). Because these groups overlapped and differed substantially in size, the estimates were considered exploratory rather than direct comparisons between ICI classes.

Era-stratified analyses showed that HBV met the criterion only in 2021–2026Q1 (ROR 1.24, 95% CI 1.04–1.47); no other viral outcome met the criterion in any of the three eras (Figure 5B; Supplementary Table S3). Several era-specific estimates were based on few events.

Comparator selection also affected the findings. When the analysis was restricted to reports with a malignant cancer indication, the overall HBV estimate no longer met the criterion (ROR 0.81, 95% CI 0.71–0.94) (Figure 5C; Supplementary Table S3). Estimates for HSV and VZV changed when broad outcome definitions were replaced with strict definitions based on small numbers of events (Supplementary Figure S2 and Table S3). No viral outcome met the reporting criterion consistently across all exposure groups or reporting eras.

### 2.6. Protocol Criteria Showed Different Temporal Patterns

The registry dataset comprised 196 trial–virus records from 164 unique trials: 51 for HBV, 18 for HCV, 38 for HIV, 83 for EBV, and 6 for CMV (Supplementary Figure S3 and Table S6). For HBV, HCV, and HIV, protocol language commonly addressed eligibility, antiviral treatment, viral-load thresholds, or immune status requirements. EBV records more often concerned EBV-associated cancers, disease context, or biomarker use.

Temporal patterns differed across the four protocol criteria when unique trials were grouped by start era (Figure 5D; Supplementary Table S6). Conditional eligibility was specified in approximately half of trials in each era (49.0–57.1%), as was antiviral treatment or prophylaxis (42.9–53.6%). Explicit viral exclusion increased from 25.0% in 2011–2015 to 35.7% in 2021–2026. Longitudinal monitoring remained uncommon, rising from 12.5% to 19.6% before declining to 8.2% in the most recent era. The four criteria therefore did not show a common temporal pattern. These findings describe protocol requirements and do not indicate how individual patients were screened or managed.

### 2.7. EBV DNA Endpoint Roles Differed Across Disease Settings

**Figure 6:**
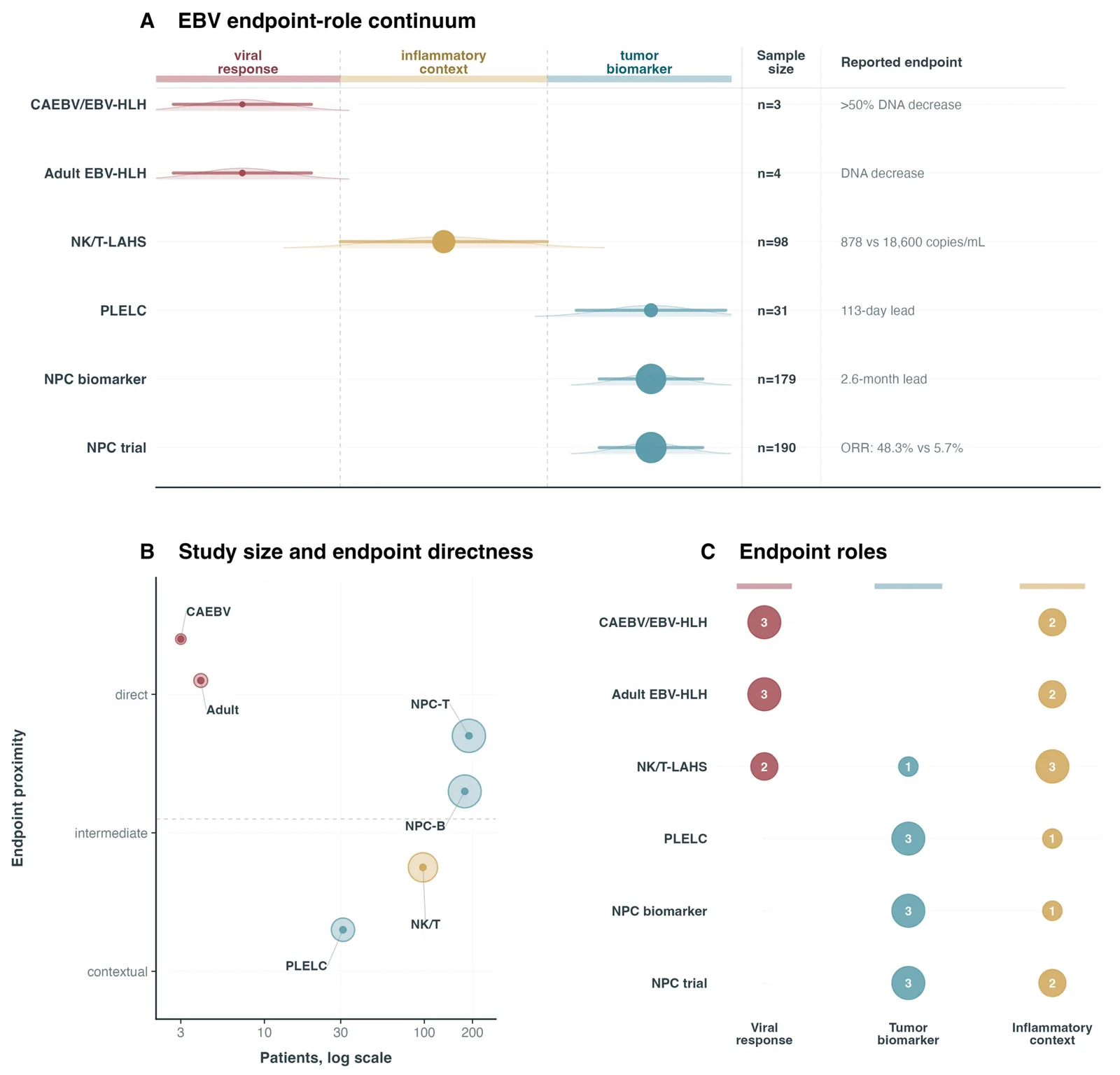
Context-dependent roles of EBV endpoints during ICI-containing therapy. (**A**) Six verified studies arranged along a descriptive continuum from viral disease response through inflammatory context to tumour burden biomarker use. Point area represents study sample size; adjacent columns report sample size and the principal EBV endpoint. Horizontal position, intervals, and ridge shapes summarize endpoint role and are not statistical effect estimates. Dashed vertical lines separate the three descriptive endpoint-role regions. (**B**) Study sample size on a logarithmic scale plotted against a descriptive classification of endpoint proximity, ranging from contextual to direct. Bubble area also represents sample size, and labels identify individual studies. The dashed horizontal line marks the midpoint of the descriptive endpoint-proximity scale. (**C**) Endpoint-role profiles across viral response, tumour biomarker, and inflammatory context. Values of 1, 2, and 3 indicate secondary or contextual, supportive, and dominant roles, respectively; zero-valued roles are not displayed. The continuum, proximity categories, and role values are author-defined descriptive classifications rather than validated evidence grades, risk-of-bias scores, causal scores, or pooled effects. Depending on the disease setting and study objective, EBV DNA may represent viral disease activity, inflammatory or lymphoproliferative activity, or tumour burden and should not be interpreted uniformly as ICI-associated viral reactivation.

Endpoint verification classified the six EBV studies into three primary roles: two evaluated EBV DNA as a direct viral disease response endpoint, one evaluated it mainly in relation to inflammatory or lymphoproliferative disease activity, and three used it primarily as a tumour burden or treatment response biomarker (Figure 6A). The primary role was closely related to disease setting: direct viral response endpoints occurred in CAEBV or EBV-HLH, the inflammatory role occurred in NK/T cell lymphoma-associated HLH, and tumour biomarker roles occurred in pulmonary lymphoepithelioma-like carcinoma or nasopharyngeal carcinoma.

The two studies with direct viral response endpoints included three and four patients, whereas studies with inflammatory or tumour biomarker roles included 31–190 patients (Figure 6B). Role-based coding further showed that EBV DNA could have both a primary and a secondary contextual role within the same study (Figure 6C; Supplementary Table S2). Across the verified studies, EBV DNA therefore represented different measured outcomes rather than a uniform reactivation endpoint.

### 2.8. Virus-Specific Findings Across Data Sources

FAERS provided a common reporting framework for the seven viral outcomes. HBV was the only outcome to meet the overall reporting criterion (226 reports; ROR 1.20, 95% CI 1.06–1.37), whereas HCV, HIV, EBV, CMV, HSV, and VZV did not (Figure 2C). The HBV estimate no longer met the criterion when the comparator was restricted to reports with malignant cancer indications (ROR 0.81, 95% CI 0.71–0.94) (Figure 5C). Estimates for HSV and VZV also varied when broad outcome definitions were replaced with strict definitions based on small event counts (Supplementary Figure S2).

The complementary analyses identified patterns that were not captured by the overall disproportionality estimates. Among 156 CMV reports, 48 (30.8%) also contained enterocolitis, pneumonitis, or both (Figure 4A,B). EBV endpoint verification identified two studies in which EBV DNA was used as a direct viral disease response endpoint, one in which it primarily reflected inflammatory or lymphoproliferative disease activity, and three in which it was primarily a marker of tumour burden or treatment response (Figure 6A–C).

The mapped literature and trial protocols also differed by viral context. HBV-related evidence frequently addressed screening, antiviral treatment or prophylaxis, and longitudinal monitoring. HCV- and HIV-related evidence more commonly addressed baseline viral control, continued antiviral treatment, and treatment eligibility (Supplementary Tables S1 and S6). FAERS quantified reporting disproportionality and same report overlap, whereas the literature and registry analyses characterized endpoint use and protocol practice.

## 3. Discussion

The principal contribution of this study is not evidence for a uniform pattern of virological events during ICI therapy, but a demonstration that findings arising during treatment have different clinical meanings. A positive test or changing viral load may indicate loss of viral control, a stable background infection, a tumour-related biomarker, or infection overlapping with immune-mediated toxicity. HBV was the only outcome to meet the overall FAERS reporting criterion, and even this modest estimate was sensitive to comparator selection; the remaining viral outcomes showed no consistent pattern across ICI classes or reporting eras. Together with the organ-specific co-reporting and endpoint verification analyses, these findings show why virological results should be interpreted in relation to baseline status, the presenting syndrome, recent immunosuppression, and the purpose of testing. This contextual approach may avoid unnecessary disruption of anticancer therapy while reducing delays in the recognition of infection, immune-mediated toxicity, or their coexistence.

Checkpoint blockade can enhance T cell activity, but release of PD-1- or CTLA-4-mediated inhibition does not necessarily produce complete or durable virological control. Responses to PD-1 blockade arise predominantly from progenitor-like exhausted T cells, whereas terminally exhausted populations have limited capacity for sustained reinvigoration; exhaustion-associated epigenetic states may also persist in HCV- and HIV-specific CD8+ T cells after antigen burden is reduced [7,8,9,25]. T cell responsiveness alone is insufficient. Effective antiviral immunity additionally requires coordinated antigen presentation, lymphocyte support, innate effector activity, and humoral immunity [10,26,27,28,29], all conditioned by viral antigen burden, infected cell lineage, anatomical distribution, and the local tissue environment. In people with HIV and cancer, reduction in the HIV reservoir after anti-PD-1 therapy was associated with pre-existing myeloid interferon and Toll-like receptor programmes and coordinated monocyte–T cell responses rather than uniform T cell expansion [30]. The virological effect of ICI therapy therefore depends on alignment among a responsive T cell compartment, a supportive immune network, and a tissue environment capable of sustaining effective antiviral immunity. Failure at any level may dissociate immune activation from virological control and tissue injury, helping to explain the heterogeneity across viruses and organs and why concurrent viral detection and inflammation do not establish either loss or restoration of viral control.

Viral detection during ICI therapy does not, by itself, indicate that checkpoint blockade has directly caused loss of virological control. At least three pathways are plausible: infection or reactivation may arise independently and resemble an irAE; ICI-induced inflammation may disrupt mucosal or alveolar barriers and alter local susceptibility; or immunosuppressive treatment for a severe or refractory irAE may permit infection to emerge downstream. CMV-associated colitis illustrates this complexity. High-dose corticosteroid initiation has been associated with infection-related hospitalisation among patients receiving PD-1 or PD-L1 inhibitors [31], and a recent two-centre cohort identified tissue-confirmed CMV in 21 of 249 patients with ICI colitis, with associations with prior infliximab exposure and greater endoscopic severity [32]. CMV may therefore mimic immune-mediated colitis, coexist with severe mucosal inflammation, or emerge after escalation of immunosuppression. The co-reporting of CMV with enterocolitis and pneumonitis in our analysis localises the organ settings in which this uncertainty may be most consequential. FAERS cannot establish whether infection preceded inflammation, mimicked an irAE, occurred concurrently, or followed irAE-directed immunosuppression because it lacks reliable onset dates, treatment sequences, serial viral measurements, and tissue findings. Co-reporting should therefore be interpreted as diagnostic overlap rather than a temporal or mechanistic relationship. Persistent or worsening enterocolitis or pneumonitis should prompt renewed evaluation for infection before immunosuppression is intensified, using longitudinal virological, imaging, endoscopic, and tissue evidence as clinically appropriate.

Whereas CMV primarily illustrates diagnostic overlap with organ toxicity, HBV raises a different question: whether a pharmacovigilance signal represents treatment-related loss of viral control. HBV was the only viral outcome to meet the prespecified overall FAERS reporting criterion, but the magnitude of disproportionality was modest, and the estimate fell below unity with the oncology-restricted comparator. This sensitivity to the reporting background argues against treating the overall signal as evidence that ICI exposure independently causes HBV reactivation. HBV is assessed more consistently than many other latent or chronic infections, with pretreatment serology, serial HBV DNA and liver biochemical testing, and antiviral management when indicated. In ICI-treated populations that include patients with HBV-related hepatocellular carcinoma, concomitant locoregional or systemic therapy and heightened clinical awareness further increase the opportunity for virological or hepatic events to be detected and reported. The attenuation with the oncology-restricted comparator is therefore compatible with surveillance effects, confounding by cancer indication and concomitant therapy, and stimulated reporting, although genuine reactivation events may also contribute.

The biological and clinical effects of checkpoint blockade on HBV are unlikely to be unidirectional. Reinvigoration of HBV-responsive T cells could improve immune control, yet the same response could intensify intrahepatic inflammation without clearing infection; subsequent corticosteroid treatment for suspected immune-mediated hepatitis may then weaken antiviral control. HBV DNA elevation, aminotransferase elevation, and clinical hepatitis are therefore related but non-equivalent outcomes. Recent studies reinforce this management-dependent interpretation. In a 2024 cohort of 1277 patients with non-small-cell lung cancer, the two reactivation events occurred in patients with chronic HBV who were not receiving antiviral therapy [33]. By contrast, no reactivation or HBV-associated hepatitis occurred among 30 patients with active HBV replication who began entecavir with durvalumab, with or without tremelimumab, in a 2025 phase 2 trial [34]. A prospective study of 1015 HBsAg-positive patients receiving concurrent antiviral therapy likewise found no significant difference in reactivation between lower and higher baseline HBV DNA groups [35]. Other recent studies have shown preserved viral control with occasional reactivation or declines in HBV markers in selected settings [35,36,37,38,39,40]. Collectively, these data indicate that baseline virological status, antiviral management, and concomitant therapy substantially modify the observed course.

The principal implication of the HBV signal is therefore not that chronic HBV infection should preclude ICI therapy. Virological status should instead be defined before treatment and distinguished from hepatic immune toxicity during follow-up. Baseline HBV serology and HBV DNA testing, antiviral prophylaxis or treatment when indicated, and longitudinal assessment of HBV DNA and liver biochemistry remain central [16,17]. When liver tests become abnormal, viral replication should be evaluated alongside immune-mediated hepatitis, tumour-related liver injury, and toxicity from concomitant treatment before ICI therapy is interrupted or immunosuppression is intensified.

For non-HBV viruses, the absence of stable disproportionality shifts interpretation from population-level signal detection to patient-level viral control, assay purpose, and evidential precision. For HCV and HIV, the principal objective during ICI therapy is usually to preserve established virological control. In a prospective study of 52 patients with HCV infection and solid tumours, four experienced a decline in HCV RNA, whereas both reactivation events occurred during immunosuppressive treatment for irAEs [41]. In the 2024 national prospective OncoVIHAC cohort, ICI therapy was feasible in people with HIV receiving antiretroviral therapy, although a CD4 count below 200 cells/µL was associated with severe irAEs [42]. HCV RNA, HIV RNA, and CD4 counts provide complementary information on viral control and immune reserve; isolated changes should be interpreted in conjunction with antiviral adherence, hepatic or immunological status, concomitant therapy, and recent immunosuppression. The FAERS findings therefore do not establish protection and should not displace individualised assessment of vulnerable patients.

EBV poses a different problem because the same laboratory measurement may represent distinct biological processes. The endpoint verification analysis showed that EBV DNA reflected virus-driven inflammatory or lymphoproliferative disease activity in CAEBV and EBV-HLH [23,43,44,45,46], whereas in nasopharyngeal and other EBV-associated cancers it was used primarily as a marker of tumour burden or treatment response [24,47,48]. An increase in EBV DNA therefore cannot be classified as loss of viral control without reference to the underlying disease, and a decline cannot automatically be interpreted as restoration of antiviral immunity. The specimen tested, the infected cell lineage, and the intended clinical use of the assay should be established before the result is used to alter ICI treatment. Whereas EBV illustrates ambiguity in endpoint meaning, HSV and VZV illustrate uncertainty arising from sparse data. Both remain clinically relevant latent infections, particularly after exposure to corticosteroids or other immunosuppressive treatment, but strict outcome definitions yielded few reports and estimates were sensitive to MedDRA term selection. Suspected HSV or VZV disease should therefore be assessed from the clinical syndrome, anatomical distribution, virological testing, and recent immunosuppressive exposure rather than inferred from an aggregate pharmacovigilance estimate. CMV occupies the separate diagnostic overlap pathway discussed above. Taken together, the non-HBV findings support longitudinal control for HCV and HIV, disease-specific endpoint interpretation for EBV, and clinical recognition despite imprecise population-level estimates for HSV and VZV.

Combination treatment modifies the clinical setting in which virological events are detected, but the relevant pathways differ by regimen. CTLA-4-containing ICI combinations may produce broader immune activation and a greater burden of immune-mediated toxicity than PD-(L)1 monotherapy [11]. Their effect on viral complications may therefore be indirect, mediated through tissue inflammation, barrier disruption, and subsequent corticosteroid or biologic immunosuppression rather than through uniform loss of antiviral immunity. In our exploratory FAERS analysis, selected HBV and CMV estimates met the reporting criterion in CTLA-4-containing groups; however, overlapping exposure definitions, unequal report counts, and absent treatment chronology preclude comparisons between regimens or assignment of the findings to an individual checkpoint inhibitor.

Other combinations introduce different sources of susceptibility and diagnostic ambiguity. Cytotoxic chemotherapy may contribute through myelosuppression, lymphopenia, mucosal injury, corticosteroid premedication, and overlapping organ toxicity. Radiotherapy may cause site-specific tissue injury and lymphocyte depletion; thoracic irradiation is particularly relevant because radiation pneumonitis, ICI pneumonitis, and pulmonary infection may have overlapping clinical and radiological features [49]. Targeted therapies are too heterogeneous to constitute a single risk category, but individual agents may produce hepatic, gastrointestinal, pulmonary, or haematological effects that resemble viral disease, modify host defence, or complicate irAE assessment. These considerations extend the clinical interpretation of our findings but were not evaluated as independent treatment effects in the present analyses. Virological findings during combination therapy should therefore be interpreted in relation to the complete regimen, treatment sequence, irradiated anatomical field, recent irAE-directed immunosuppression, antiviral prophylaxis, and baseline viral status rather than assigned to the ICI component alone.

The findings support staged assessment based on virological status and the presenting syndrome, rather than exclusion from ICI therapy solely because a chronic or latent infection is present. Before treatment, the first question is whether infection is clinically active and uncontrolled. Previous exposure, infection without active replication, or chronic infection that remains stable with appropriate antiviral therapy should not, by itself, justify delaying ICI treatment. Pretreatment evaluation should instead prioritise uncontrolled replication with progressive organ injury, active systemic infection, or circumstances in which necessary antiviral treatment and monitoring cannot yet be implemented. Its purpose is to identify patients who may benefit from antiviral intervention or intensified surveillance, rather than to exclude all virus-positive patients [16,17]. During treatment, virological testing should address a defined clinical question: HBV DNA helps prevent reactivation and interpret hepatic injury; HCV RNA, HIV RNA, and CD4 counts assess continued viral control and immune reserve; EBV DNA requires disease-specific interpretation; CMV testing is most informative in severe, atypical, or immunosuppression-refractory inflammation; and HSV or VZV testing should be syndrome-driven. When hepatitis, enterocolitis, or pneumonitis develops, the task is not to force a binary choice between infection and an irAE, but to reconstruct the event sequence and consider coexistence. Serial viral measurements, recent corticosteroid or biologic exposure, imaging, endoscopy, and tissue findings should be integrated with alternative explanations, including tumour progression, concomitant anticancer therapy, other drug-induced injury, and co-pathogens. A single positive viral result does not establish causation, just as an irAE-compatible syndrome does not exclude infection.

The assessment should lead to a management decision rather than merely a diagnostic label. ICI therapy may often continue with monitoring when virological control is stable, and there is no clinical evidence of infection-related organ injury. Active replication or tissue-invasive disease should prompt pathogen-directed treatment and consideration of temporary ICI interruption according to severity. When infection and an irAE are both plausible, response to initial corticosteroids should not be the sole basis for determining the cause, and the evidence for infection should be reassessed before immunosuppression is intensified. Multidisciplinary review may be valuable in complex cases. This framework does not impose a uniform withholding threshold; it aims to reduce unnecessary interruption of effective anticancer therapy while limiting two opposing errors—escalating immunosuppression after infection has been misclassified as an irAE, and delaying treatment of immune-mediated inflammation after viral detection has been mistaken for causal infection.

A strength of this study is its coverage of the clinical ICI era from early adoption to contemporary practice, using data from 2011Q1 to 2026Q1 to examine reporting estimates and protocol practices across changing treatment and surveillance environments. Trial-era analyses showed no uniform temporal relaxation of viral eligibility or monitoring criteria, suggesting that contemporary protocol handling remains heterogeneous rather than following a single progressive trend. The layered design kept pharmacovigilance signals, clinical evidence, endpoint verification, and protocol mapping analytically distinct so that convergence and discordance remained visible rather than being collapsed into a composite score. This extends prior syntheses focused on efficacy and safety in viral hepatitis-associated hepatocellular carcinoma by addressing how virological findings are interpreted when they arise during treatment [50,51,52]. Future trials and real-world datasets should record baseline viral status, antiviral treatment or prophylaxis, longitudinal viral measurements, ICI class and combination therapy, cancer type, organ domain toxicity, irAE-directed immunosuppression, and the intended clinical role of each assay.

Several limitations define the boundaries of these conclusions. FAERS lacks treatment denominators, exposure duration, baseline viral status, antiviral prophylaxis, reliable event chronology, cancer stage detail, and complete information on corticosteroid or biologic immunosuppression. It is also vulnerable to duplicate clinical concepts, notoriety effects, surveillance bias, stimulated reporting, and residual confounding by indication, cancer type, disease severity, and concomitant therapy [53,54,55]. The oncology-restricted comparator reduces but does not remove these differences, and neither disproportionality nor same report overlap can establish incidence, protection, event sequence, mechanism, or causality. The ICI-class and CTLA-4-containing combination analyses were exploratory; overlapping exposure groups, unequal report counts, and the very small dual-primary suspect combination subset precluded comparative inference between regimens. The other evidence layers introduce different constraints: the literature map is susceptible to publication bias, heterogeneous outcome definitions, incomplete verification outside the study-level evidence set, and unequal precision across designs, while registry classifications reflect recorded protocol text rather than amendments, site-level decisions, adherence, or patient outcomes. A common certainty grade cannot validly equate case reports, observational cohorts, trials, and pharmacovigilance studies, and assigning evidence to prevention, monitoring, endpoint interpretation, or infectious mimic roles required clinical judgement despite prespecified definitions. The findings should therefore be interpreted as a structured framework rather than as estimates of incidence, comparative risk, or causality. Within these limits, the framework identifies where virological findings can inform prevention, monitoring, or diagnostic reassessment, and where stronger longitudinal evidence remains necessary.

## 4. Materials and Methods

### 4.1. Study Design

We conducted a multi-source evaluation of virological events during ICI therapy using four complementary components: FAERS disproportionality analyses, PubMed evidence mapping with design-specific study appraisal, focused verification of EBV endpoint roles, and assessment of virus-related eligibility and monitoring criteria in ClinicalTrials.gov (Table 1). These components addressed three related questions: whether broad viral outcomes were disproportionately reported with ICIs; in which settings viral events overlapped with immune–inflammatory organ toxicity; and how virological findings were interpreted or managed in published studies and clinical trial protocols. Each source was analysed separately according to its unit of analysis and evidentiary scope. Findings were then compared across sources to identify virus- and context-specific patterns relevant to prevention, monitoring, and diagnostic reassessment. The study was not designed to estimate incidence or comparative treatment risk, or to establish temporal or causal relationships.

### 4.2. FAERS Data Source and Exposure Definition

We analysed FAERS quarterly ASCII files from 2011Q1 through 2026Q1 [53]. This interval covered reporting from early ICI adoption to the most recent complete quarter available at data extraction. Quarterly DEMO files were combined, and records were grouped by CASEID. For each CASEID, the record with the highest CASEVERSION was retained; when records shared the same highest version, the highest PRIMARYID was used as a deterministic tiebreaker. CASEIDs listed in the quarterly DELETE files were excluded before the analytic datasets were constructed. DRUG, REAC, and INDI records were then restricted to the retained PRIMARYIDs. This procedure yielded 17,898,429 deduplicated reports.

The primary ICI cohort comprised reports in which a PD-1, PD-L1, or CTLA-4 inhibitor was designated as the primary suspect. Exploratory analyses evaluated PD-1, PD-L1, and CTLA-4 primary suspect groups separately, as well as CTLA-4-containing combinations in which a PD-(L)1 or CTLA-4 inhibitor was recorded as a primary or secondary suspect. These groups could overlap and were not intended for direct treatment comparisons. A stricter definition requiring both agents to be designated as primary suspects identified only eight reports and was not analysed further. Primary analyses were not stratified by cancer type because FAERS indication fields were incomplete and heterogeneous, precluding reliable cancer-specific comparisons. Drug dictionaries and operational definitions are provided in Supplementary Table S7.

### 4.3. Outcome Definitions

Viral outcomes were grouped into seven clinically interpretable categories: HBV reactivation, HCV flare, HIV viral instability, and EBV, CMV, HSV, and VZV infection or reactivation. The rationale and MedDRA definitions for each category are provided in Supplementary Tables S7 and S8. Because HSV and VZV reports were sparse and sensitive to term selection, stricter reactivation definitions were examined in sensitivity analyses (Supplementary Table S3). FAERS does not consistently record baseline viral load, antiviral treatment, immune status, or longitudinal follow-up; these categories therefore represent reported viral events rather than confirmed incident loss of virological control.

Immune–inflammatory outcomes were selected to identify organ toxicities that may be clinically difficult to distinguish from viral disease. They comprised hepatic toxicity, viral hepatitis/liver injury, enterocolitis, pneumonitis, myocarditis, myositis, neuromuscular toxicity, nephritis, and endocrinopathy. This analysis was restricted to 181,500 reports listing a PD-1 or PD-L1 inhibitor as the primary suspect and was conducted separately from the 200,699-report viral outcome analysis. Outcome definitions, estimates, and organ domain assignments are provided in Supplementary Table S4. These outcomes were used to define potential diagnostic overlap settings, not as presumed causes of viral events.

### 4.4. FAERS Disproportionality Analysis

For each exposure–outcome pair, we constructed a two-by-two contingency table comparing primary suspect ICI reports with all other deduplicated FAERS reports. Reporting odds ratios (RORs) and 95% confidence intervals were calculated on the log scale [54,55,56]. The prespecified reporting criterion required at least three exposed event reports and a lower 95% confidence limit above 1.0.

### 4.5. Virus–Organ Same Report Overlap

We assessed same report overlap between viral outcomes and organ domain immune–inflammatory outcomes in the primary suspect ICI cohort. For each virus–organ pair, we calculated the number and proportion of reports with the viral outcome that also contained the organ domain outcome (Supplementary Table S5). Prespecified clinical settings included hepatic events with HBV or HCV and enterocolitis or pneumonitis with CMV. The analysis described coding overlap within individual reports; it did not assess event order, infection after immunosuppressive treatment, mechanism, or causality.

### 4.6. Sensitivity Analyses

Robustness was examined across ICI exposure groups, reporting eras, comparator populations, and outcome definitions. The temporal analysis used three non-overlapping intervals—2011–2015, 2016–2020, and 2021–2026Q1—to represent early adoption, expanding use, and contemporary practice while retaining sufficient reports for virus-specific estimation. Comparator sensitivity was assessed among reports with mapped malignant cancer indications by comparing 171,592 ICI reports with 2,413,268 reports without recognized ICI primary or secondary suspect exposure. HSV and VZV estimates were additionally tested using stricter reactivation definitions. These analyses assessed the stability of reporting patterns; they did not estimate incidence or comparative treatment effects, and RORs below 1.0 were not interpreted as protective.

### 4.7. PubMed Evidence Mapping

PubMed was searched from database inception through 23 June 2026 for human clinical evidence on therapeutic ICI exposure in the seven viral contexts. The main multivirus search identified 1424 unique records, and a focused HSV/VZV update applied the same eligibility criteria. The PMID was used for deduplication and screening. Eligible records reported viral control or loss of control, viral load kinetics, flare or reactivation, antiviral management, viral context safety, tumour-related biomarker use, immune–inflammatory overlap, or trial design implications. We excluded preclinical studies, records without therapeutic ICI exposure, incidental viral mentions without an eligible endpoint, ineligible publication types, and oncolytic or vector studies without a natural infection endpoint.

Rule-assisted screening was used to prioritise candidate records and audit exclusions. Candidate records and potentially inconsistent exclusions then underwent manual title-and-abstract adjudication, with full-text examination when required and available. Eligible publications were assigned either to descriptive evidence volume mapping or to the study-level evidence set. Publication counts described evidence volume rather than effect magnitude or certainty. Search strategies, screening counts, exclusion reasons, eligibility rules, and publication-level decisions are provided in Supplementary Table S1.

Study-level publications underwent design-specific appraisal using the relevant JBI checklist, RoB 2, ROBINS-I, or an adapted pharmacovigilance reporting appraisal. Abstract-only or incompletely verified records contributed only to descriptive mapping. Because the appraisal tools addressed different study designs, their judgements were not combined into a numerical score or common certainty grade (Supplementary Table S2). Within the study-level evidence set, EBV studies reporting EBV DNA, EBV-associated disease activity, tumour response, or inflammatory outcomes during ICI-containing treatment underwent focused endpoint-role verification.

### 4.8. ClinicalTrials.gov Trial Landscape Assessment

ClinicalTrials.gov was searched through 23 June 2026 to evaluate how ICI trial protocols addressed HBV, HCV, HIV, EBV, and CMV [57]. These viruses could be represented in protocols through baseline serostatus, viral-load or immune status thresholds, antiviral treatment or prophylaxis, longitudinal monitoring, or biomarker endpoints. HSV and VZV, which are more often recorded as episodic clinical events than as standardized protocol variables, were evaluated in FAERS and PubMed but were outside the prespecified registry scope.

Records were analysed at the trial–virus level, allowing one trial to contribute to more than one viral context; analyses based on unique trials were used for sensitivity assessment. Extracted criteria included explicit exclusion, conditional or managed eligibility, antiviral treatment or prophylaxis, viral-load or CD4 thresholds, and longitudinal monitoring. Keyword-assisted classification was verified against supporting registry text. Search strategies, extraction fields, trial characteristics, and sensitivity analyses are provided in Supplementary Table S6.

Trials were grouped by start year as pre-2011, 2011–2015, 2016–2020, or 2021–2026. The latter three intervals matched the FAERS reporting eras and represented early, expanding, and contemporary ICI use. Two pre-2011 trials were retained in the overall mapping but were not displayed as a separate era because the stratum was too small for meaningful comparison. Era-specific proportions were reported with 95% Wilson confidence intervals.

### 4.9. Cross-Source Evaluation

Cross-source comparison followed a structured sequence. Operational definitions were established before the final comparison, and no numerical weighting or composite score was applied. For each virus, we first examined the overall FAERS estimate and its stability across exposure groups, reporting eras, comparator populations, and outcome definitions. We then compared these reporting patterns with virus–organ same report overlap, publication-level endpoint roles, design-specific appraisal, focused EBV endpoint verification, and registry protocol criteria. Evidence was considered convergent when sources supported the same clinical interpretation and discordant when the direction, stability, or meaning of the findings differed. The resulting roles—targeted prevention, contextual monitoring, endpoint interpretation, and infectious mimic evaluation—describe potential clinical uses of the evidence; they are not evidence grades, causal classifications, or comparative risk scores. Operational rules are provided in Supplementary Table S7.

### 4.10. Reporting and Statistical Software

Reporting followed relevant STROBE and RECORD-PE principles where applicable [58,59]. Data management and statistical analyses were performed using Python version 3.13.7 and R version 4.4.2. Analytic definitions, source tables, and reproducible outputs are provided in the Supplementary Files.

## 5. Conclusions

Among the viral outcomes evaluated, HBV was the only outcome to meet the prespecified FAERS reporting criterion, although the modest signal was sensitive to comparator selection. Evidence from the literature and trial registries further indicated that virological findings during ICI therapy have context-dependent clinical meanings, ranging from baseline viral status and treatment continuity to disease-specific biomarkers, infectious mimics of immune-related adverse events, and infections arising after immunosuppressive treatment. Accordingly, a controlled chronic or latent viral infection should not, by itself, preclude ICI therapy. When virological abnormalities or organ inflammation emerge, clinical decisions should integrate event chronology, serial viral measurements, antiviral treatment, recent immunosuppressive exposure, and organ-specific evidence before interrupting ICI therapy or escalating immunosuppression. Taken together, this virus- and context-specific framework may support more precise prevention, monitoring, and diagnostic reassessment, helping clinicians avoid both unnecessary interruption of ICI therapy and delayed recognition of infection or immune-mediated toxicity.

## Figures and Tables

**Table 1:** Data sources, analytic roles, and interpretive scope.

Data Source and Analysis	Dataset and Period	Analytic Scope	Analytic Contribution	Interpretive Scope
FAERS primary analysis	17,898,429 deduplicated reports, including 200,699 primary suspect ICI reports; 2011Q1–2026Q1	Primary suspect PD-1, PD-L1, or CTLA-4 inhibitor exposure; seven viral outcomes, prespecified immune–inflammatory outcomes, and virus–organ same report overlap	Report counts, RORs with 95% CIs, and descriptive coding overlap	Characterizes relative reporting patterns, not incidence, absolute risk, patient-level viral status, event sequence, mechanism, or causality
FAERS oncology-restricted comparator analysis	171,592 ICI reports and 2,413,268 non-ICI reports with malignant cancer indications; 2011Q1–2026Q1	Same exposure and viral outcome definitions, with the comparator restricted to malignant cancer reports	Tests the sensitivity of ROR estimates to the background reporting population	Reduces but does not remove confounding by indication, cancer type, disease severity, treatment selection, surveillance, prophylaxis, or reporting practice
PubMed evidence map	1424 unique search records; 198 mapped publications; database inception to 23 June 2026	Human clinical evidence on ICI therapy across seven viral contexts	Describes evidence volume, study design, sample size, and reported endpoint roles	Does not provide pooled incidence or effect estimates, causal inference, or equal evidential weight across heterogeneous designs
Study-level evidence appraisal	84 full-text-verified or full-text-adjudicated publications; through 23 June 2026	Design-specific extraction and appraisal of populations, ICI regimens, viral status, virological endpoints, clinical outcomes, and safety context	Verifies endpoint roles and identifies design-specific methodological limitations	Does not provide a cross-design numerical quality score, common certainty grade, or pooled causal effect
ClinicalTrials.gov landscape and trial start era analysis	196 trial–virus records from 164 unique trials; registry records available through 23 June 2026	Protocol criteria for viral exclusion, conditional eligibility, antiviral treatment or prophylaxis, and longitudinal monitoring	Describes protocol handling and variation across trial start eras	Does not establish patient-level eligibility decisions, protocol adherence, virological outcomes, treatment safety, or causal effects
EBV endpoint verification	Six verified ICI-containing studies; publications available through 23 June 2026	EBV DNA and related endpoints across viral, inflammatory or lymphoproliferative, and tumour-associated disease settings	Distinguishes viral disease response, inflammatory activity, and tumour burden biomarker roles	Does not estimate generalizable EBV reactivation incidence, establish an ICI effect, or support pooled comparison across endpoint roles

Abbreviations: CI, confidence interval; CTLA-4, cytotoxic T-lymphocyte-associated protein 4; EBV, Epstein–Barr virus; FAERS, FDA Adverse Event Reporting System; ICI, immune checkpoint inhibitor; PD-1, programmed cell death protein 1; PD-L1, programmed death-ligand 1; ROR, reporting odds ratio. Note: Data sources were analysed separately and compared across sources without numerical weighting or a composite causal score. Their analytic roles are complementary rather than interchangeable.

## Data Availability

Aggregated analysis outputs, figure source data, and audit files are included in the Supplementary Files. Raw FAERS quarterly data are publicly available from the US FDA FAERS database, ClinicalTrials.gov records are publicly available from ClinicalTrials.gov, and PubMed records are publicly searchable through the National Library of Medicine. Analysis scripts and derived source-data tables are provided with the submission package or are available from the corresponding authors upon request.

## Supplementary Materials

The following supporting information can be downloaded at: https://www.mdpi.com/article/10.3390/ph19101616/s1, Supplementary Figure S1. Cancer-indication composition and coding completeness in ICI reports with viral outcomes. Supplementary Figure S2. Sensitivity of HSV and VZV estimates to outcome definition. Supplementary Figure S3. ClinicalTrials.gov viral-context landscape and protocol characteristics. Supplementary Table S1. PubMed screening, eligibility, and publication-level evidence map. Supplementary Table S2. Design-specific quality assessment and endpoint verification. Supplementary Table S3. Complete virus-specific FAERS disproportionality analyses. Supplementary Table S4. Immune-inflammatory reporting estimates and organ-domain mapping. Supplementary Table S5. Virus–organ same-report overlap and cancer-indication analyses. Supplementary Table S6. ClinicalTrials.gov trial characteristics and trial-start-era analyses. Supplementary Table S7. FAERS exposure, comparator, outcome, and analysis definitions. Supplementary Table S8. Prespecified viral scope and clinical rationale.
