# Supplementary material for: Virological Events During Immune Checkpoint Inhibitor Therapy: A Multi-Source Evaluation
Source: Pharmaceuticals (Basel). 2026 Oct 12;19(10):1616. doi: 10.3390/ph19101616 (PMC13708036; doi:10.3390/ph19101616)
Supplement: Supplementary file 1 [file pharmaceuticals-19-01616-s001.zip › Supplementary_Figure_Legends.pdf]

Supplementary Figure Legends

Supplementary Figure S1. Cancer-indication composition and coding completeness in ICI reports with viral outcomes.
(A) Concentration of specifically mapped cancer indications by viral outcome. Red points represent the largest cancer group, blue points represent the combined proportion of the three largest groups, and connecting lines show the difference between these measures. Calculations were restricted to reports assigned to one specific cancer group; cancer not otherwise specified and multiple or ambiguous indications were excluded. (B) Proportion of reports with each viral outcome that contained any mapped cancer indication. Labels show the number of mapped reports divided by the total number of reports for that viral outcome. (C) Distribution of mutually exclusive cancer-indication categories. Nonspecific, multiple, and ambiguous indications are grouped as other or unspecified cancers. Percentages are displayed for viral outcomes with at least 40 mapped reports and for categories comprising at least 18% of that viral group. These analyses describe indication composition and coding completeness; they do not estimate cancer-specific incidence or comparative viral risk.

Supplementary Figure S2. Sensitivity of HSV and VZV estimates to outcome definition.
(A) Composition of the prespecified MedDRA preferred-term sets. Strict definitions contain only explicit reactivation terms; broad definitions additionally include prespecified infection and organ-disease terms; and the extended VZV definition further includes varicella-specific terms. Numbers at the ends of the bars indicate the total number of preferred terms in each definition. (B) Reporting odds ratios and 95% confidence intervals for the corresponding definitions, using all other deduplicated FAERS reports as the comparator. Filled symbols denote estimates based on at least 10 exposed-event reports, whereas open symbols denote sparse estimates based on fewer than 10 reports; symbol fill does not indicate whether the prespecified reporting criterion was met. The vertical dashed line marks a reporting odds ratio of 1.0. These analyses assess sensitivity to outcome coding rather than incidence or causality; estimates below 1.0 do not imply protection, and sparse estimates should be interpreted cautiously.

Supplementary Figure S3. ClinicalTrials.gov viral-context landscape and protocol characteristics.
(A) Distribution of 196 trial–virus records across HBV, HCV, HIV, EBV, and CMV, stratified as direct viral trials, ICI trials with viral context, or context-only trials. Direct viral trials evaluated a virus or virus-associated condition as a central intervention or outcome; ICI trials with viral context explicitly incorporated viral status or management; context-only trials recorded the virus as a disease or biomarker context without a direct viral objective. (B) Proportion of trial–virus records containing prespecified registry-text features. Bubble area and colour intensity represent the same proportion. (C) Target-enrolment distribution among records with available enrolment data. Point shape represents trial context, point colour represents registry status, and horizontal bars show the within-virus interquartile range. (D) Registry-status composition and (E) trial-phase composition within each viral context. The 196 trial–virus records arose from 164 unique trials, and one trial could contribute to more than one viral context. HSV and VZV were outside the prespecified

registry scope. These analyses describe recorded protocol characteristics rather than patient-level eligibility decisions, protocol adherence, virological outcomes, treatment safety, or causal effects.

Abbreviations: CI, confidence interval; CMV, cytomegalovirus; EBV, Epstein-Barr virus; FAERS, FDA Adverse Event Reporting System; HBV, hepatitis B virus; HCV, hepatitis C virus; HIV, human immunodeficiency virus; HSV, herpes simplex virus; ICI, immune checkpoint inhibitor; MedDRA, Medical Dictionary for Regulatory Activities; ROR, reporting odds ratio; VZV, varicella-zoster virus.
