# Supplementary material for: Virological Events During Immune Checkpoint Inhibitor Therapy: A Multi-Source Evaluation
Source: Pharmaceuticals (Basel). 2026 Oct 12;19(10):1616. doi: 10.3390/ph19101616 (PMC13708036; doi:10.3390/ph19101616)
Supplement: Supplementary file 1 [file pharmaceuticals-19-01616-s001.zip › Supplementary_Table_Legends.pdf]

Legends for Supplementary Tables

Supplementary Table S1. PubMed screening, eligibility, and publication-level evidence map.
(A) Screening and evidence-map assembly, including consolidated primary reasons for exclusion and assignment to the study-level evidence set or descriptive evidence mapping. (B) Characteristics and endpoint roles of the 198 unique mapped publications. (C) Eligibility, classification, and evidence-use rules. (D) Virus-specific composition of the mapped literature by analytic tier. The evidence map comprised 189 eligible publications from the prespecified 1,424-record search and nine PMID-deduplicated publications identified in the focused HSV/VZV update. Publications addressing more than one virus contribute to multiple virus-specific categories; these counts therefore should not be summed to derive the number of unique publications.

Supplementary Table S2. Design-specific quality assessment and endpoint verification.
(A) Design-specific quality or risk-of-bias assessments for the 84 publications in the study-level evidence set. (B) Abstract-only publications retained for descriptive evidence mapping. (C) Verification of the clinical roles assigned to EBV endpoints. (D) Appraisal outcomes summarized by assessment framework. Because different tools were used for different study designs, their judgements were not combined into a numerical score or common certainty scale. Six full-text-verified EBV studies are presented in Figure 6; two additional abstract-only studies were retained in the table but were not formally appraised.

Supplementary Table S3. Complete virus-specific FAERS disproportionality analyses.
(A) Estimates for the overall primary-suspect ICI cohort and exploratory ICI exposure groups. (B) Estimates across three non-overlapping FAERS reporting eras. (C) Sensitivity analysis using reports with malignant-cancer indications and no recognized ICI exposure as the comparator. (D) Sensitivity analyses using alternative HSV and VZV outcome definitions. The strict primary-suspect intersection of PD-(L)1 and CTLA-4 therapy contained eight reports and no viral events; its continuity-corrected estimates are provided only for completeness. RORs quantify reporting disproportionality and should not be interpreted as incidence, absolute risk, treatment effects, or causality.

Supplementary Table S4. Immune-inflammatory reporting estimates and organ-domain mapping.
(A) Disproportionality estimates for prespecified immune-inflammatory outcomes in the 181,500-report PD-1/PD-L1 primary-suspect cohort. (B) Mapping of these outcomes to descriptive organ domains. The analyses provide immune-inflammatory reporting context for evaluating potential diagnostic overlap with viral events. Organ-domain summaries may include more than one outcome from the same report.

Supplementary Table S5. Virus–organ same-report overlap and cancer-indication analyses.
(A) Complete matrix of seven viral outcomes and eight immune-inflammatory outcomes in the 200,699-report ICI cohort. (B) Focused analyses of HBV with hepatic events and CMV with enterocolitis or pneumonitis. (C) Cancer-indication mapping and coding audit. Same-report overlap identifies co-recorded

events but does not establish their sequence or causal relationship. Cancer groups may overlap and do not provide cancer-specific denominators or risk estimates.

Supplementary Table S6. ClinicalTrials.gov trial characteristics and trial-start-era analyses.
(A) Trial–virus records, start year, trial context, eligibility handling, and virus-related protocol criteria. (B) Protocol criteria according to trial-start era and virus. (C) Sensitivity analysis using unique trials as the analytic unit. (D) Sensitivity analysis according to trial context. The dataset contains 196 trial–virus records from 164 unique trials. Protocol criteria were classified using reproducible keyword-assisted review of the supporting registry text. These data describe protocol design and do not represent patient-level eligibility decisions, management, or outcomes.

Supplementary Table S7. FAERS exposure, comparator, outcome, and analysis definitions.
(A) ICI drug dictionary used to identify PD-1, PD-L1, and CTLA-4 agents in normalized FAERS drug fields. (B) MedDRA preferred terms used to define viral and immune-inflammatory outcomes, including broad, strict, and extended HSV/VZV definitions. (C) Operational rules for exposure groups, comparators, reporting eras, signal detection, and cross-source evaluation. Exposure groups may overlap and should not be interpreted as head-to-head treatment comparisons. The prespecified reporting criterion required at least three exposed-event reports and a lower 95% confidence limit of the ROR greater than 1.0.

Supplementary Table S8. Prespecified viral scope and clinical rationale.
This table defines the seven viral contexts included in the study and summarizes their biological states, relevant virological endpoints, and clinical interpretive roles. The framework distinguishes chronic or latent infection, loss of viral control, infectious mimics, inflammatory or lymphoproliferative activity, and tumour-associated viral biomarkers.

Abbreviations: CAEBV, chronic active Epstein-Barr virus infection; CI, confidence interval; CMV, cytomegalovirus; EBV, Epstein-Barr virus; FAERS, FDA Adverse Event Reporting System; HBV, hepatitis B virus; HCV, hepatitis C virus; HIV, human immunodeficiency virus; HLH, haemophagocytic lymphohistiocytosis; HSV, herpes simplex virus; ICI, immune checkpoint inhibitor; irAE, immune-related adverse event; MedDRA, Medical Dictionary for Regulatory Activities; NCT, ClinicalTrials.gov identifier; PD-1, programmed cell death protein 1; PD-L1, programmed death-ligand 1; PRR, proportional reporting ratio; PS, primary suspect; ROR, reporting odds ratio; SS, secondary suspect; VZV, varicella-zoster virus.
